# PTBP1 suppresses porcine epidemic diarrhea virus replication *via* inducing protein degradation and IFN production

**DOI:** 10.1016/j.jbc.2023.104987

**Published:** 2023-06-29

**Authors:** Wenzhen Qin, Ning Kong, Yu Zhang, Chunmei Wang, Sujie Dong, Huanjie Zhai, Xueying Zhai, Xinyu Yang, Chenqian Ye, Manqing Ye, Wu Tong, Changlong Liu, Lingxue Yu, Hao Zheng, Hai Yu, Wen Zhang, Daoliang Lan, Guangzhi Tong, Tongling Shan

**Affiliations:** 1 Shanghai Veterinary Research Institute, Chinese Academy of Agricultural Sciences, Shanghai, China; 2 Jiangsu Co-innovation Center for Prevention and Control of Important Animal Infectious Diseases and Zoonoses, Yangzhou University, Yangzhou, China; 3 College of Animal & Verterinary Sciences, Southwest Minzu University, Chengdu, China; 4 Department of Preventive Dentistry, Shanghai Ninth People’s Hospital, College of Stomatology, Shanghai Jiao Tong University School of Medicine, Shanghai, China; 5 School of Medicine, Jiangsu University, Zhenjiang, China

**Keywords:** PEDV, PTBP1, N protein, IFN-I, selective autophagy

## Abstract

Porcine epidemic diarrhea virus (PEDV) causes severe morbidity and mortality among newborn piglets. It significantly threatens the porcine industry in China and around the globe. To accelerate the developmental pace of drugs or vaccines against PEDV, a deeper understanding of the interaction between viral proteins and host factors is crucial. The RNA-binding protein, polypyrimidine tract–binding protein 1 (PTBP1), is crucial for controlling RNA metabolism and biological processes. The present work focused on exploring the effect of PTBP1 on PEDV replication. PTBP1 was upregulated during PEDV infection. The PEDV nucleocapsid (N) protein was degraded through the autophagic and proteasomal degradation pathways. Moreover, PTBP1 recruits MARCH8 (an E3 ubiquitin ligase) and NDP52 (a cargo receptor) for N protein catalysis and degradation through selective autophagy. Furthermore, PTBP1 induces the host innate antiviral response *via* upregulating the expression of MyD88, which then regulates TNF receptor–associated factor 3/ TNF receptor–associated factor 6 expression and induces the phosphorylation of TBK1 and IFN regulatory factor 3. These processes activate the type Ⅰ IFN signaling pathway to antagonize PEDV replication. Collectively, this work illustrates a new mechanism related to PTBP1-induced viral restriction, where PTBP1 degrades the viral N protein and induces type Ⅰ IFN production to suppress PEDV replication.

Porcine epidemic diarrhea virus (PEDV) is a member of the genus *Alphacoronavirus* from the Coronaviridae family. It causes acute porcine epidemic diarrhea, an infectious bowel disorder with high contagiousness, leading to significant financial losses in the porcine industry. Porcine epidemic diarrhea is manifested as piglet dehydration, diarrhea, and vomiting, and it presents a mortality rate of up to 100% (1, 2). Vaccination is currently highly efficient in controlling infectious disorders. However, the existing commercial vaccines are not adequate to protect against the epidemic strains. Viruses interact or hijack various host factors during the infection to accomplish virus replication. The nucleocapsid (N) protein is the main structural protein of PEDV and is expressed in the highest abundance during virus infection (3). Previous studies have elucidated that the N protein of PEDV interacts with host factors like EGR1 (4), PABPC4 (5), TRIM21 (6), and BST2 (7). Moreover, the N protein plays a protective role in helping the host against PEDV *via* enhancing autophagy and activating the interferon (IFN) pathway. Typically, inherent immunity in the host is predominantly accomplished by the IFN pathway and represents the first line of defense against pathogenic microorganisms (8, 9). The PEDV N protein inhibits *in vitro* type I/III IFN responses for facilitating virus replication (10, 11). Moreover, the N protein exerts an essential effect on counteracting the host’s inherent immunity by inhibiting IFN expression (11, 12).

Autophagy is one of the major intracellular degradation systems. The aggregated cytosolic components or organelles are delivered to and degraded in autolysosomes in eukaryotic cells. Mechanisms of autophagy include three patterns: macroautophagy/autophagy, microautophagy, and chaperone-mediated autophagy (13, 14). Upon virus infection, host autophagy provides the intrinsic antiviral defense and contributes to the inhibition of viral replication. Examples include the Sindbis virus, human parainfluenza virus type 3, and vesicular stomatitis virus (15, 16, 17). Gassen *et al*. clarified that inhibition of the S-phase kinase-associated protein 2 promotes autophagy and reduces Middle East respiratory syndrome coronavirus replication (18). Interestingly, some viral species, including influenza A virus and hepatitis B virus, develop strategies to escape autophagic degradation and even utilize autophagy mechanisms to enhance their viral replication and pathogenesis (19, 20, 21, 22, 23, 24). An in-depth understanding of PEDV proteins and host factor interaction will facilitate further knowledge regarding the pathogenic mechanisms of PEDV.

Polypyrimidine tract–binding proteins (PTBPs) are members of the heterogeneous nuclear ribonucleoproteins subfamily, and they include RNA-binding proteins together with the heterogeneous nuclear RNA complex (25, 26). PTBPs are related to nuclear pre-mRNAs and affect their processing, mRNA transport, and metabolism. PTBP1, one of the PTBPs, represses exonal inclusion and modulates alternative exonal screening during mRNA processing (26). PTBP1 is the nuclear factor responsible for alternative splicing and participates in posttranscriptional regulation, RNA localization, and mRNA stability when it shuttles in the cytoplasm (27, 28). A recent study showed that miR-326 upregulated the autophagy of olfactory mucosal mesenchymal stem cells by the PI3K pathway by targeting PTBP1 (29). PTBP1 also promoted breast cancer cell proliferation by autophagy and the PTEN/Akt pathway (30).

This study aimed to elucidate the mechanism by which PTBP1 regulates PEDV infection. PTBP1 was observed to target and degrade the virus N protein and inhibit PEDV replication. Moreover, PTBP1 activated the type Ⅰ IFN signaling pathway by upregulating the expression of myeloid differentiation factor 88 (MyD88).

## Results

### PTBP1 level decreased after PEDV infection

**Figure 1:**
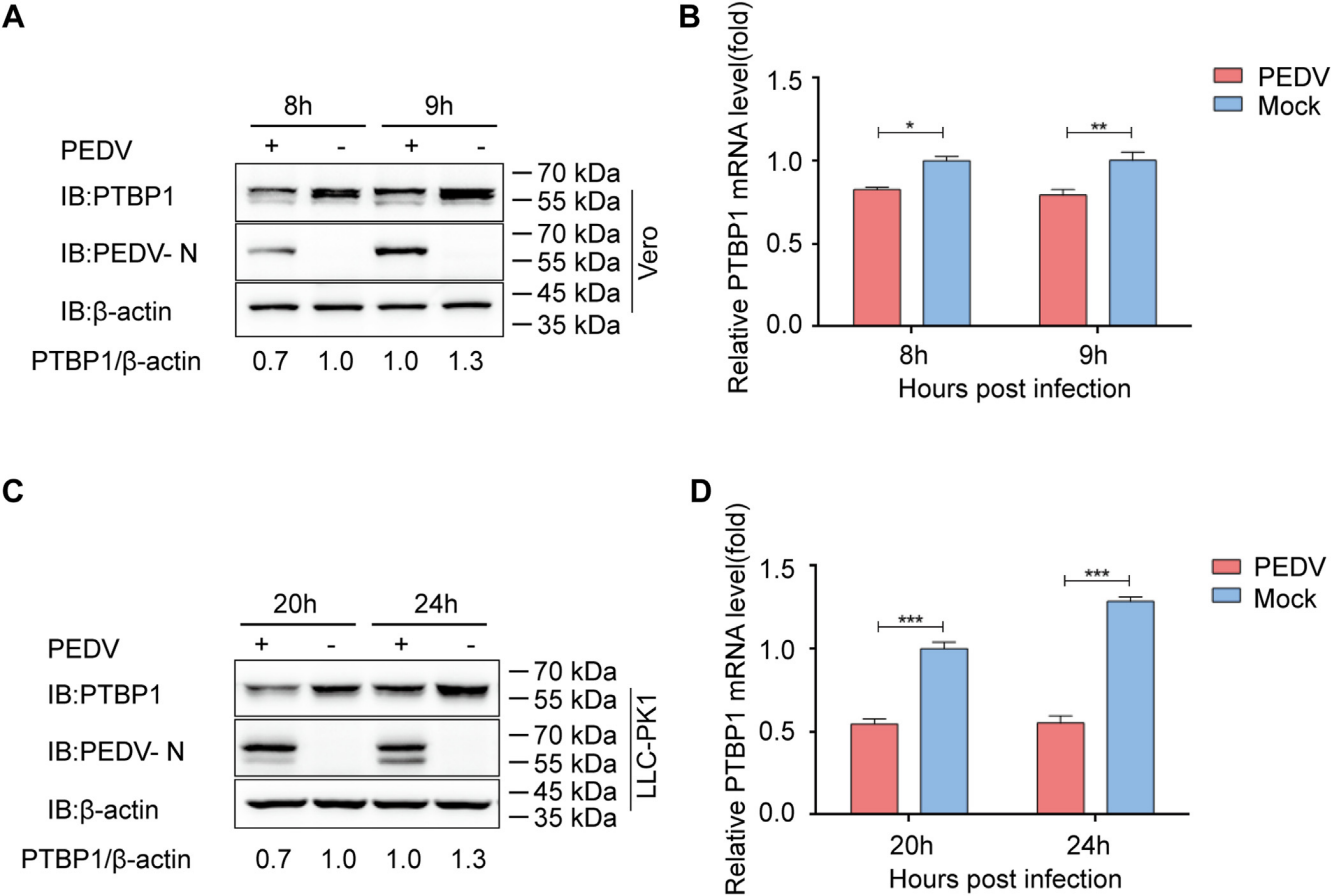
**PTBP1 expression is downregulated during PEDV replication.***A*, PEDV (MOI = 1) with or without infection in Vero cells, followed by analysis at 8 and 9 hpi. The protein expression was detected through Western blot assay. *B*, the mRNA expression of PTBP1 within identical samples (*A*) was measured through qRT-PCR. *C*, PEDV (MOI = 1) with or without infection in LLC-PK1 at 20 and 24 hpi. The protein expression was detected through the Western blot assay. *D*, the mRNA expression of PTBP1 within identical samples (*C*) was measured through qRT-PCR. Results indicate means ± SD from three samples. ∗*p* < 0.05, ∗∗*p* < 0.01, and ∗∗∗*p* < 0.001 upon Student’s *t* test (two-sided). MOI, multiplicity of infection; PEDV, porcine epidemic diarrhea virus; PK1, porcine kidney 1; PTBP, polypyrimidine tract–binding protein; qRT-PCR, quantitative real-time PCR.

To screen the potential antiviral proteins–regulated PEDV replication, we performed co-immunoprecipitation (co-IP) assay to search for the proteins that interacted with PEDV N protein, and the N-affinity–isolation assay was analyzed by mass spectrometry. A lot of proteins were identified to be interacting with PEDV N, including PTBP1 protein. The possible role of PTBP1 in antiviral responses was investigated by studying the involvement of PEDV infection on the cellular PTBP1 expression. The PEDV (strain JS-2013)-infected Vero cells (multiplicity of infection, MOI = 1) were harvested for quantitative real-time PCR (qRT-PCR) and Western blot assays, according to a previous report (31). Endogenous PTBP1 was significantly downregulated in the Vero cells infected by PEDV than in the noninfected counterparts (Fig. 1, *A* and *B*). Moreover, the expression of PTBP1 was confirmed in PEDV-infected LLC-porcine kidney 1 (PK1) cells at 20 and 24 h post infection (hpi) (Fig. 1, *C* and *D*). These results showed that the PTBP1 level had decreased after PEDV infection.

### PTBP1 inhibits PEDV replication

**Figure 2:**
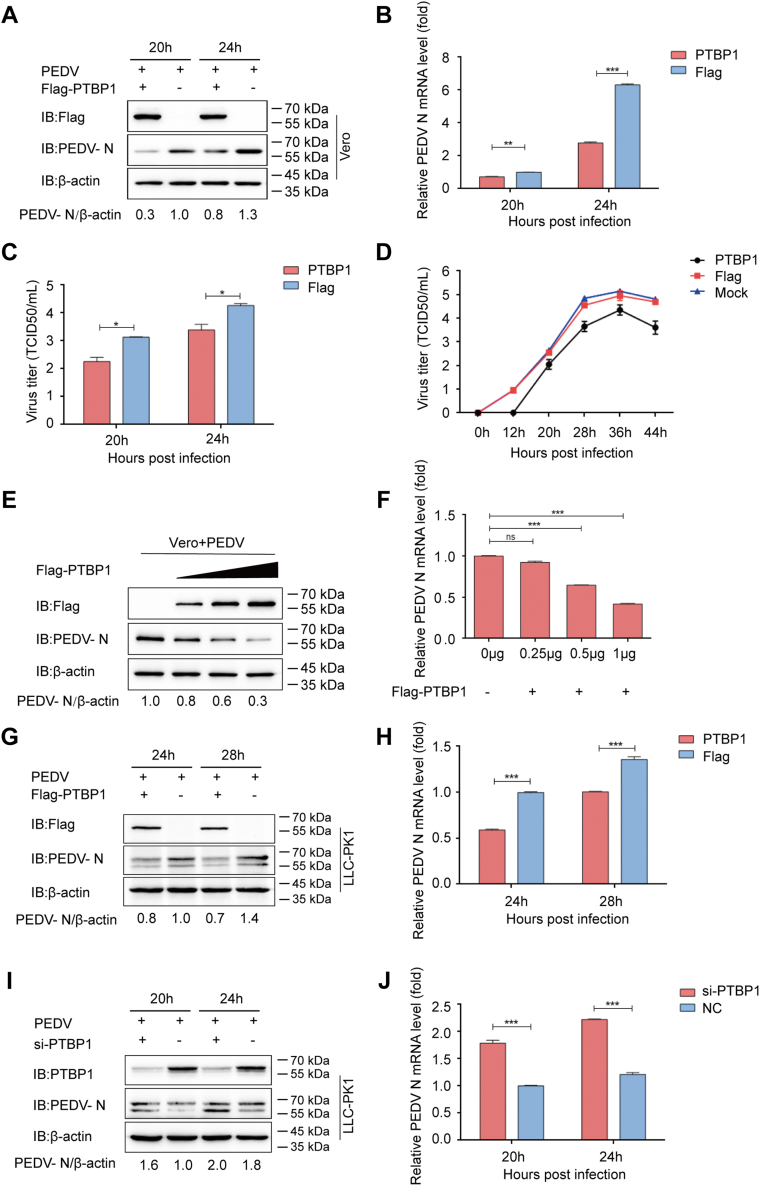
**PEDV replication is inhibited by PTBP1.***A*–*C*, PTBP1 plasmid transfection and PEDV infection at MOI = 0.01 was conducted in Vero cells. PEDV replication was analyzed with Western blot, qRT-PCR, and TCID_50_. *D*, Flag-PTBP1 plasmid transfection and PEDV infection at MOI = 0.01 were conducted in Vero cells. The culture supernatant was collected at the indicated time points to detect TCID_50_ that denoted viral titers. *E* and *F*, elevating doses of Flag-PTBP1 plasmid transfection and PEDV infection at MOI = 0.01 were conducted in Vero cells. qRT-PCR and Western blot assays were performed to analyze the cell supernatants and lysates. *G* and *H*, PTBP1 plasmid transfection along with PEDV infection was conducted in the LLC-PK1 cells. qRT-PCR and Western blot assays were conducted to analyze PEDV replication. *I* and *J*, PTBP1 siRNA or NC siRNA was transfected in the LLC-PK1 cells. After 24 h of transfection, the cells were infected with PEDV. qRT-PCR and Western blot assays were conducted to examine PEDV replication. Results are denoted as means ± SD from three samples. ∗*p* < 0.05, ∗∗*p* < 0.01, and ∗∗∗*p* < 0.001 upon Student’s *t* test (two-sided). MOI, multiplicity of infection; PEDV, porcine epidemic diarrhea virus; PK1, porcine kidney 1; PTBP, polypyrimidine tract–binding protein; qRT-PCR, quantitative real-time PCR.

For exploring the mechanism of action of PTBP1 on PEDV infection, Vero cells were transfected with the PTBP1 plasmids (Flag-PTBP1) and then infected with PEDV (MOI = 0.01) 24 hpi. After that, the infected cells were harvested along with their corresponding supernatants. The viral loads and PEDV N expression were detected through qRT-PCR, Western blot, and median tissue culture infectious dose (TCID_50_). The results showed that PTBP1 significantly inhibited PEDV replication in the Vero cells (Fig. 2, *A*–*C*). The viral titers in the Vero cells overexpressing PTBP1 had reduced considerably compared with the empty vector–transfected counterparts (Fig. 2*D*). The proliferation efficiency of PEDV significantly decreased with an increase in the PTBP1 plasmid dose (Fig. 2, *E* and *F*). Similarly, PEDV N mRNA and protein expression declined within the PEDV-challenged LLC-PK1 cells (Fig. 2, *G* and *H*). Further, silencing the expression of PTBP1 increased the PEDV replication (Fig. 2, *I* and *J*). Therefore, PEDV replication was inhibited by PTBP1.

### PTBP1 targets and degrades PEDV N protein

**Figure 3:**
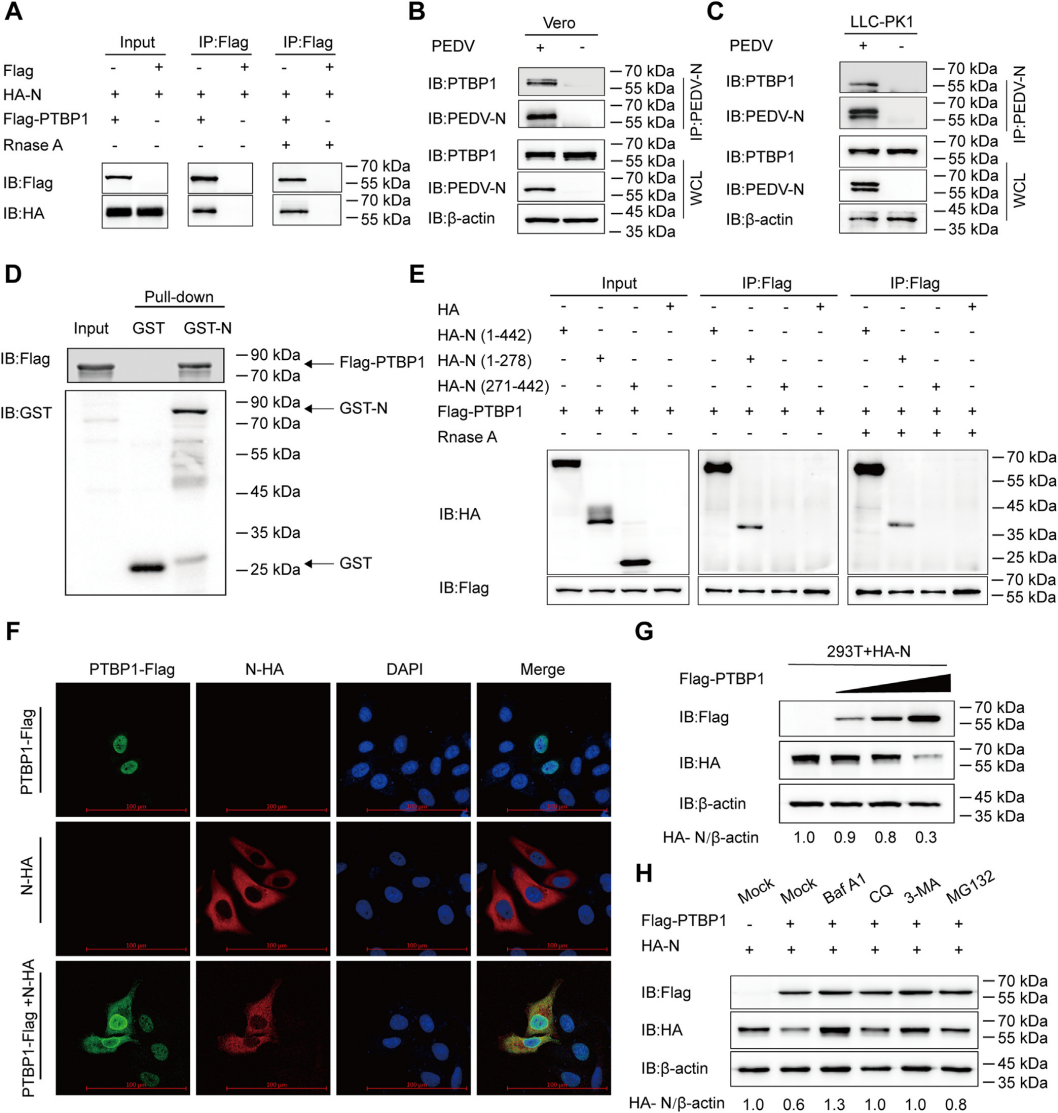
**PTBP1 targets and degrades PEDV N protein.***A*, plasmids that encoded HA-N and Flag-PTBP1 were transfected in HEK 293T cells for 24 h; the co-IP assay was conducted using the anti-Flag–binding beads. The protein expression was examined through Western blot assay. The interaction between Flag-PTBP1 and PEDV N protein was also detected following RNase exposure. *B*, Vero cells with or without PEDV infection (MOI = 0.01). Later, the cells were collected to immunoprecipitate the endogenous PTBP1 with an anti-PEDV N protein antibody. *C*, LLC-PK1 cells with or without PEDV infection (MOI = 0.1). The cells were collected to immunoprecipitate the endogenous PTBP1 with an anti-PEDV N protein antibody. *D*, expression of PEDV N and PTBP1 was induced within the BL21 bacterial strain (DE3) to perform the GST affinity–isolation assay. Eluted protein expression was examined through Western blot assay. *E*, co-IP assay of 293T cells transfected with N or the indicated N mutants, together with a vector encoding Flag-PTBP1. *F*, plasmids that encoded N-HA and Flag-PTBP1 were transfected into the HeLa cells for 24 h, followed by labeling using specific primary antibodies and secondary antibodies. DAPI was utilized for nuclear staining, and a confocal immunofluorescence microscope was utilized to observe fluorescence signals. The scale bars represent 100 μm. *G*, plasmids that encoded HA-N and elevated doses of Flag-PTBP1 were cotransfected into HEK 293T cells for 24 h. Western blot assay was conducted for analyzing the cell lysates. *H*, plasmids that encoded HA-N and Flag-PTBP1 were cotransfected into the HEK 293T cells, followed by treatments using MG132, Baf A1, 3MA, and CQ, respectively. Western blot assay was conducted for analyzing the cell lysates. 3MA, 3-methyladenine; Baf A1, bafilomycin A1; Co-IP, co-immunoprecipitation; CQ, chloroquine; GST, glutathione-*S*-transferase; HEK, human embryonic kidney; MOI, multiplicity of infection; PEDV, porcine epidemic diarrhea virus; PK1, porcine kidney 1; PTBP, polypyrimidine tract–binding protein.

The interaction of PTBP1 with the structural proteins (E, M, N, and S) of PEDV was investigated. The co-IP assay showed Flag-PTBP1–mediated PEDV N precipitation; the binding was not interfered by cell lysis with RNase in human embryonic kidney (HEK) 293T cells (Fig. 3*A*). Furthermore, co-IP showed that the N protein of PEDV effectively co-immunoprecipitated with the internal PTBP1 protein (Fig. 3, *B* and *C*). The glutathione-*S*-transferase (GST) affinity–isolation assay confirmed the direct binding between PEDV N protein and PTBP1 (Fig. 3*D*). To determine the domains of PEDV N involved in its interaction with the PTBP1 protein, we constructed the deletion mutants of N (amino acids 1–278, amino acids 271–422) and cotransfected HEK 293T cells with the plasmids and a plasmid encoding the Flag-PTBP1. A co-IP analysis showed that PTBP1 interacted with the full-length N protein (amino acids 1–442) and N terminal of N protein (amino acids 1–278), whereas C terminal of N protein (amino acids 271–442) did not (Fig. 3*E*), indicating that the N terminal of N protein is important for its interaction with PTBP1. The cytoplasmic-nuclear shuttling of PTBP1 modulates RNA processes (25). To determine the binding localization of PTBP1 and PEDV N protein, plasmids encoding N-HA and PTBP1-Flag were cotransfected into HeLa cells. PTBP1 was predominantly localized in the nucleus and shuttled to the cytoplasm to efficiently colocalize with the PEDV N protein (Fig. 3*F*). This indicated that PTBP1 interacts with PEDV N protein in the cytoplasm.

To explore the effect of PTBP1 on PEDV N protein, Western blot was performed with increasing amounts of Flag-PTBP1 plasmids and HA-N plasmids in the HEK 293T cells. Additionally, the quantity of PEDV N protein decreased with an increase in the Flag-PTBP1 concentration (Fig. 3*G*). Since PTBP1 suppressed the PEDV N protein in a dose-dependent manner, it was hypothesized that PTBP1 might boost host protein degradation pathways to inhibit PEDV N protein. The autolysosomal pathway and the ubiquitin-proteasome system represent the two main pathways related to cellular protein degradation (14). HEK 293T cells were cotransfected with Flag-PTBP1 and the HA-N plasmids and then treated with the protease inhibitor MG132 and autophagy inhibitors bafilomycin A1, 3-methyladenine, and chloroquine, respectively. The PEDV N protein expression increased within the group was treated with autophagy and protease inhibitors (Fig. 3*H*). Collectively, these results suggested that PTBP1 reduced the PEDV N protein expression, and protease and autophagy inhibitors reversed the suppression.

### PTBP1 degrades PEDV N protein by activating the PTBP1–MARCH8–NDP52–autophagosome pathway

**Figure 4:**
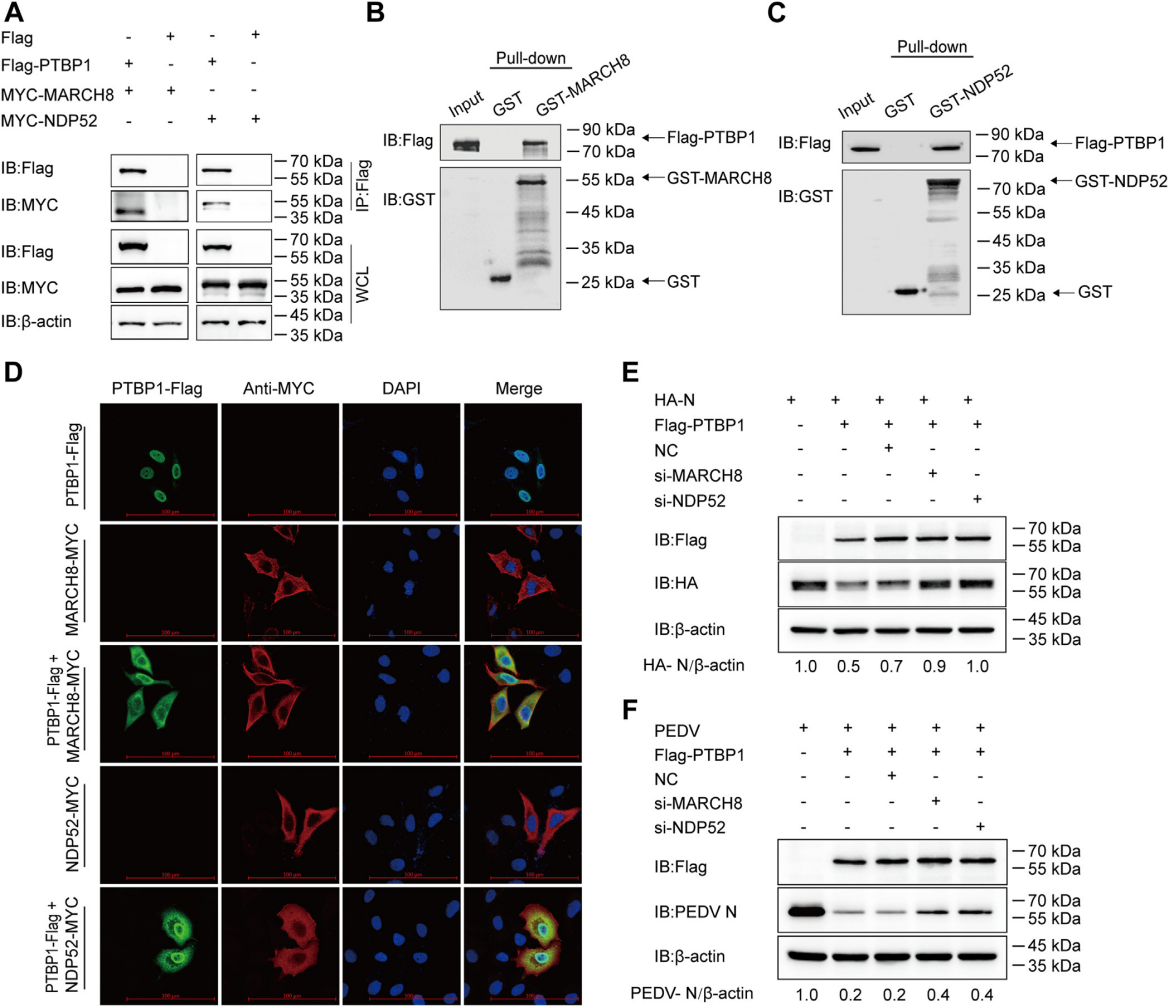
**PTBP1 contributes to PEDV N protein degradation *via* the PTBP1–MARCH8–NDP52–autophagosome pathway.***A*, plasmids encoding Flag-PTBP1, MYC-MARCH8, or MYC-NDP52 were transfected into the HEK 293T cells for 24 h. The co-IP assay was performed using anti-Flag–binding beads. Western blot assay was conducted for analyzing cell lysates. *B* and *C*, expression of PTBP1 and GST-MARCH8 or GST-NDP52 was induced within the BL21 bacterial strain (DE3), followed by purification before conducting GST affinity–isolation assay. *D*, plasmids that encoded Flag-PTBP1 and MYC-NDP52 or MYC-MARCH8 were transfected into the HeLa cells, followed by labeling using corresponding primary as well as secondary antibodies. DAPI was adopted for nuclear staining, and a confocal immunofluorescence microscope was employed to observe fluorescence signals. The scale bars represent 100 μm. *E*, plasmids that encoded iRNA (MARCH8 siRNA, or NC siRNA, or NDP52 siRNA), HA-N, and Flag-PTBP1 were transfected into the HEK 293T cells. Protein expression was measured through Western blot assay. *F*, plasmids that encoded Flag-PTBP1 and MARCH8 siRNA or NDP52 siRNA or NC siRNA were transfected in Vero cells for 24 h. Afterward, PEDV (MOI = 0.01) was infected in the cells, which were lysed to analyze PEDV N protein concentration by Western blot assay. Co-IP, co-immunoprecipitation; GST, glutathione-*S*-transferase; HEK, human embryonic kidney; MOI, multiplicity of infection; PEDV, porcine epidemic diarrhea virus; PTBP, polypyrimidine tract–binding protein.

A previous study reported that autophagy was induced by PEDV infection (7). Several host antiviral factors recruit MARCH8 (an E3 ubiquitin ligase) to catalyze PEDV N protein ubiquitination. This process is identified *via* NDP52 (a cargo receptor), which is then transmitted to and degraded in the autophagosome (5, 7). The interaction between PTBP1 and MARCH8 and NDP52 was examined using the co-IP and GST affinity-isolation assays. PTBP1 co-immunoprecipitated with MARCH8 and NDP52 in the HEK 293T cells (Fig. 4*A*). The GST affinity–isolation assay confirmed the direct binding of PTBP1 and MARCH8 or NDP52 (Fig. 4, *B* and *C*). The plasmids encoding Flag-PTBP1 and MARCH8-MYC or NDP52-MYC were cotransfected into HeLa cells to identify the colocalization of PTBP1 and MARCH8 or NDP52. The confocal immunofluorescence assay indicated that PTBP1 in the nucleus shuttled to the cytoplasm and colocalized with MARCH8 and NDP52 in the cytoplasm (Fig. 4*D*). Hence, PTBP1 could target MARCH8 and PEDV N protein to NDP52 to achieve autophagy-based protein degradation. For determining the requirement of the MARCH8-NDP52–mediated autophagy pathway in PTBP1-mediated N protein degradation, siRNAs were selected to downregulate the expression of MARCH8 or NDP52. Interrupting the expression of MARCH8 or NDP52 significantly increased the abundance of HA-N (Fig. 4*E*). The result was also confirmed in the PEDV-infected Vero cells (MOI = 0.01) at 24 hpi of PTBP1 together with MARCH8 siRNA or NDP52 siRNA (Fig. 4*F*). These findings indicated that PTBP1 induced PEDV N protein degradation *via* the MARCH8–NDP52–autophagosome pathway.

### PTBP1 induces the IFN signaling pathway to restrict PEDV infection

**Figure 5:**
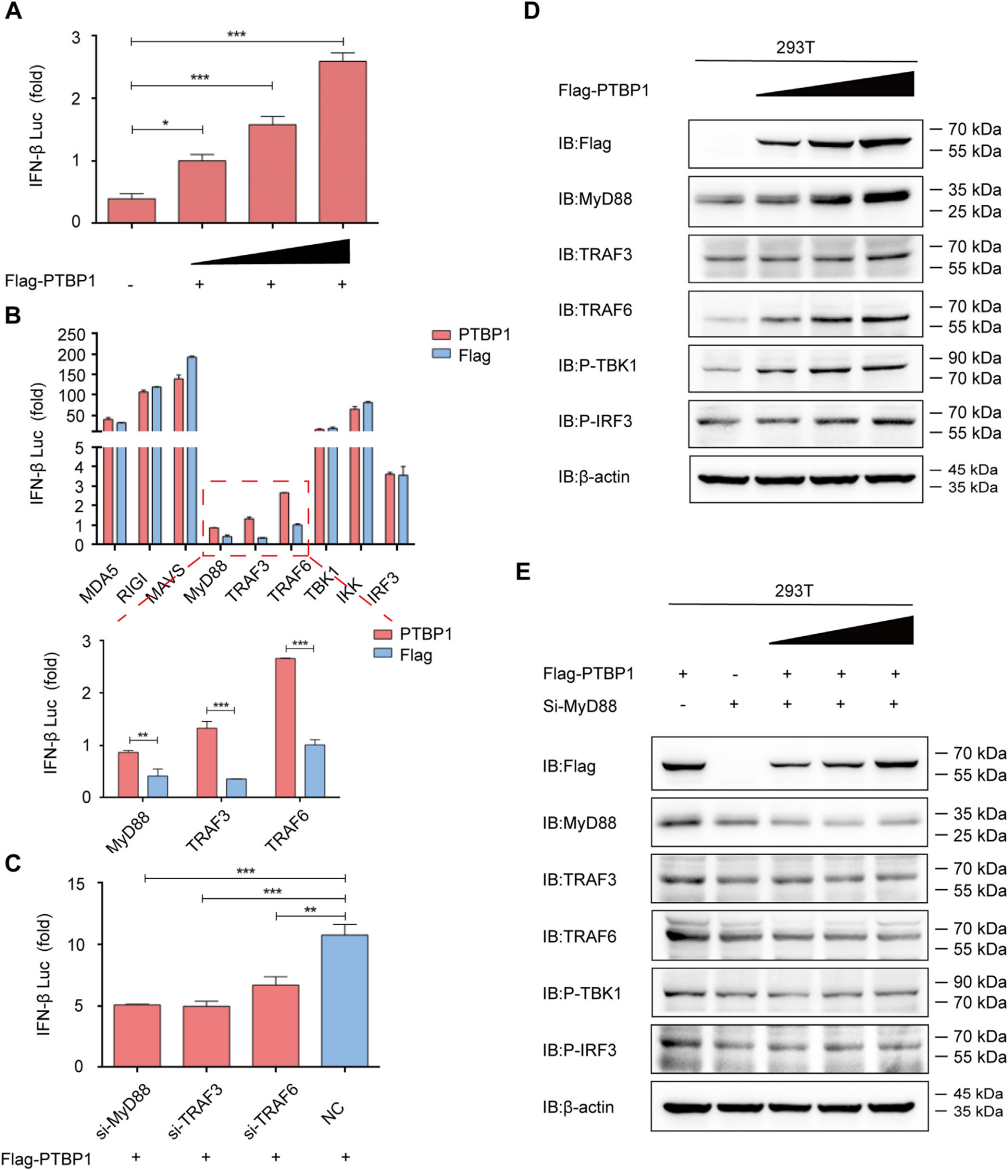
**PTBP1 induces IFN innate antiviral response.***A*, IFN-β luciferase reporter was transfected with Flag-PTBP1 at elevating doses (*wedge*) into the HEK 293T cells, and luciferase activities were examined. *B*, plasmids encoding PTBP1 and IFN-β luciferase reporter were cotransfected with plasmids encoding MDA5, RIG-I, MAVS, MyD88, TRAF3, TRAF6, TBK1, IKK, or IRF3 in the HEK 293T cells, and luciferase activities were examined. *C*, Flag-PTBP1, IFN-β luciferase reporter, and siRNA (MyD88 siRNA, TRAF3 siRNA, or TRAF6 siRNA) were cotransfected into the HEK 293T cells, and then luciferase activities were examined. *D*, Flag-PTBP1 at elevating doses (*wedge*) was transfected in the HEK 293T cells for 24 h. Western blot assay was conducted to analyze the cell lysates. *E*, Flag-PTBP1 at elevating doses (*wedge*) and MyD88 siRNA were cotransfected in HEK 293T cells for 24 h. Western blot assay was conducted to analyze the cell lysates. Data are means ± SD of triplicate samples. ∗*p* < 0.05, ∗∗*p* < 0.01, ∗∗∗*p* < 0.001, two-tailed Student’s *t* test. HEK, human embryonic kidney; IRF, IFN regulatory factor; MyD, myeloid differentiation factor; PIFN, interferon; TBP, polypyrimidine tract–binding protein; TBK, TANK– binding kinase; TRAF, TNF receptor–associated factor.

Inherent immunity is the first line of defense against virus infections in the host, preventing virus attacks. Upon viral infections, host immune cells quickly produce type I IFNs and then induce various IFN-stimulated host genes and enter an antiviral state (32, 33). The relationship between IFN and PTBP1 was investigated in the HEK 293T cells to clarify the contribution of IFN-mediated antiviral responses in antagonizing PEDV. The luciferase reporter assay showed that PTBP1 facilitated IFN-β expression in a dose-dependent manner (Fig. 5*A*). Further, the PTBP1 expression plasmids and plasmids that encoded the critical proteins were cotransfected; they were related to an inherent antiviral reaction. Overexpressed PTBP1 triggered the luciferase reporter activity induced by MyD88, TNF receptor–associated factor (TRAF)3, and TRAF6 (Fig. 5*B*). Interfering with the expression of MyD88, TRAF3, or TRAF6 blocked the IFN-β expression induced by PTBP1 (Fig. 5*C*). Flag-PTBP1 plasmids at elevating doses were transfected into HEK 293T cells to validate the impact of PTBP1 on boosting the host innate antiviral response. Resultantly, PTBP1 protein efficiently induced the expression of endogenous MyD88, TRAF6, phosphorylated IFN regulatory factor 3 (IRF3), and phosphorylated TANK–binding kinase 1 (TBK1) in a dose-dependent manner (Fig. 5*D*). The IFN-inducing function of PTBP1 was lost when the HEK 293T cells were treated with MyD88 siRNA (Fig. 5*E*). Therefore, these results showed that PEDV might be inhibited by the PTBP1-induced MyD88–TRAF3/TRAF6–TBK1–pIRF3–IFN signaling pathway.

## Discussion

**Figure 6:**
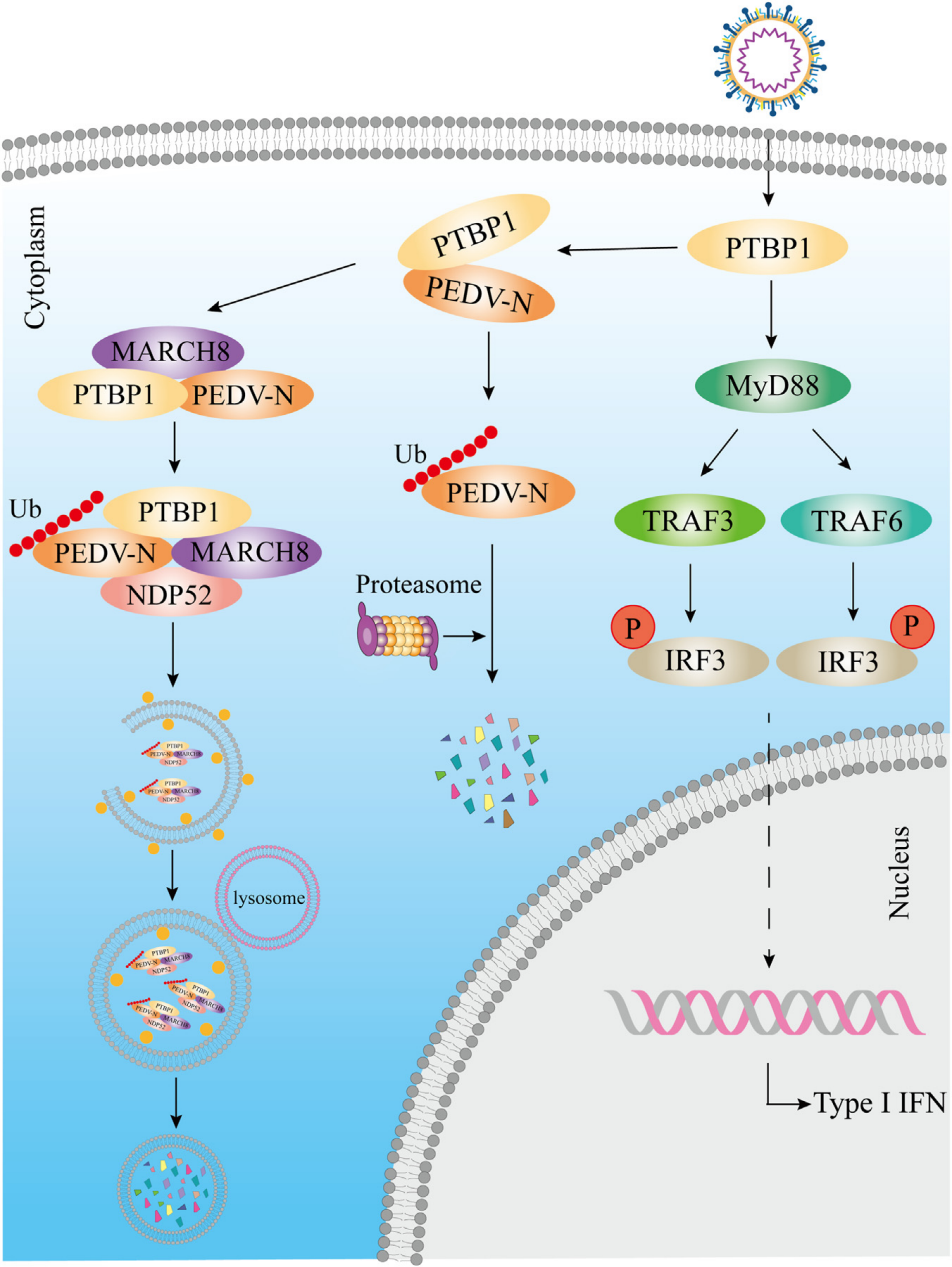
**PTBP1 inhibits PEDV replication by an antiviral mechanism**. PEDV, porcine epidemic diarrhea virus; PTBP, polypyrimidine tract–binding protein.

PEDV is a threat to the porcine industry in China and around the globe. A better understanding of the interaction between virus and host factors is essential in accelerating the speed by which drugs or vaccines against PEDV are developed. The PEDV N protein implements several functions, like the host cell cycle regulation and immune system interference, within the viral replication cycle and pathogenesis (3). The N protein combines with the viral RNA to provide the structural basis for the spiral nucleocapsid, which interacts with the M protein and is packaged into virus particles to form the virus core (34). The N protein is associated with virus replication and transcription (35) and participates in the biological processes of PEDV survival from the host immune system (7, 34). The N protein is also involved in regulating the host cell cycle to promote PEDV replication (36). This study demonstrates that the antiviral function of PTBP1 resists PEDV replication. PTBP1 induced the PEDV N protein degradation *via* the protease and MARCH8–NDP52–autophagosome pathways (Fig. 6). Moreover, PTBP1 induces the host’s innate antiviral response *via* the MyD88–TRAF3/TRAF6–TBK1–pIRF3–IFN pathway to antagonize PEDV (Fig. 6).

PTBP1 contains four RNA recognition motifs, one nuclear export signal, and a bipartite nuclear localization domain (37). It plays an important role in controlling RNA metabolism, including mRNA localization, mRNA alternative splicing, polyadenylation, translation depending on the internal ribosome entry site, and mRNA stability (38). Additionally, PTBP1 is associated with cell cycle control (39). The current study results found that endogenous PTBP1 was downregulated during PEDV infection, and overexpression of PTBP1 suppressed PEDV replication within the Vero and LLC-PK1 cells. Furthermore, PTBP1 suppressed PEDV replication through PEDV N protein targeting and degradation. Autophagy, an essential cellular process, plays a crucial role in the viral life cycle and disease pathogenesis in the game between the virus and the host. For example, the host factor S-phase kinase-associated protein 2 attenuates autophagy to promote Middle East respiratory syndrome coronavirus replication (18). Recently, PTBP1 was reported to promote breast cancer cell proliferation *via* autophagy and the PTEN/Akt pathway (30). In the present study, PTBP1 induced PEDV N protein degradation *via* the autophagy and protease pathways. During selective autophagy, the E3 ubiquitin ligase ubiquitinates the substrate proteins, which are then recognized by cargo receptors. These receptors are delivered to the ATG8 family proteins, forming autophagosome degradation substrates (40). One of our previous reports demonstrated that the host antiviral factors PABPC4 and BST2 recruited MARCH8 to catalyze PEDV N protein ubiquitination; NDP52 then recognizes the ubiquitinated virus proteins and transfers them to the lysosome for degradation (5, 7). In this study, PTBP1 mediated PEDV N protein degradation *via* the MARCH8–NDP52–autophagosome pathway. Moreover, interfering MARCH8 or NDP52 expression reversed the PTBP1-induced PEDV N protein degradation.

Innate immunity, principally type I IFN, activates the antiviral state of the host cells and resists virus replication (41). During viral infection, the pattern recognition receptors in the host identify the viral RNA while triggering the IFN response [69]. Pattern recognition receptors recruit the downstream adapter protein MAVS after detecting viral RNAs in the cytoplasm; they recruit TRAF3/TRAF6 and activate the TBK1. On the other hand, IFN-α/β is activated by phosphorylating and transferring the nuclear IRF 3/7 (42, 43, 44, 45). Here, overexpressed PTBP1 triggered the MyD88–TRAF3/TRAF6–TBK1–pIRF3–IFN innate antiviral response pathway upon PEDV infection. During PEDV infection, PTBP1 combines with MyD88, which regulates TRAF3, TRAF6, and TBK1. It further induces phosphorylated IRF3 migration into the nucleus to combine with the IFN-β promoter and activate IFN-β production. The continuous arms race between viruses and hosts has certainly driven the evolution of the host restriction factors. PEDV N protein suppresses IRF3 activation and downregulates the production of type I IFN by sequestering the interaction between IRF3 and TBK1 to facilitate PEDV replication (10). Corresponding to this, the host activates the antiviral function of PTBP1, which degrades the viral N protein and induces type Ⅰ IFN production to inhibit PEDV replication.

In summary, the regulation of PTBP1 during PEDV replication is a novel discovery. Moreover, PTBP1 is responsible for PEDV N protein degradation *via* the MARCH8–NDP52–autophagy and protease pathways. PTBP1 induces the host’s innate antiviral response *via* the MyD88–TRAF3/TRAF6–TBK1–pIRF3–IFN pathway and inhibits PEDV replication. The current study provides a new mechanism of N protein degradation and activation of the IFN signaling pathway by which PTBP1 suppresses PEDV replication.

## Experimental procedures

### Antibodies and reagents

Anti-PTBP1 antibody (catalog no. WH0005725M1), anti-DYKDDDDK-tag antibody (catalog no. 14793s), mouse anti-MYC-tag antibody (catalog no. 2276s), rabbit anti-MYC-tag antibody (catalog no. 2278s), and bafilomycin A1 (catalog no. 54645) were purchased from Cell Signaling Technology. Anti-HA antibody (catalog no. H6908), anti-Flag M2 antibody (catalog no. F1804), MG132 (catalog no. M7449), chloroquine (catalog no. PHR1258), and 3-methyladenine (catalog no. M9281) were obtained from Sigma-Aldrich. Anti-GST antibody (catalog no. 10000-0-AP), anti-ACTB/β-actin (catalog no. 66009-1-Ig), horseradish peroxidase (HRP)-conjugated anti-rabbit immunoglobulin G (IgG) antibody (catalog no. SA00001-2), and HRP-conjugated anti-mouse IgG antibody (catalog no. SA00001-1) were purchased from Proteintech Group. Alexa Fluor 488 goat anti-rabbit IgG (H+L) cross-adsorbed secondary antibody (catalog no. A-11008), Alexa Fluor 488 goat anti-mouse IgG (H+L) cross-adsorbed secondary antibody (catalog no. A-11001), Alexa Fluor 594 goat anti-rabbit IgG (H+L) cross-adsorbed secondary antibody (catalog no. A-11037), and Alexa Fluor 594 goat anti-mouse IgG (H+L) cross-adsorbed secondary antibody (catalog no. A-11032) were obtained from Invitrogen. Primers were obtained from Sangon Biotech, and siRNA were designed and purchased from GenePharma (Table 1). Human MARCH8 siRNA (catalog no. sc-90432) and human NDP52 siRNA (catalog no. 93738) were purchased from Santa Cruz Biotechnology.

**Table:** Primer and siRNA sequences used in this study

Purpose	Names	Sequence (5′-3′)
	PEDV *N* forward	GAGGGTGTTTTCTGGGTTG
	PEDV *N* reverse	CGTGAAGTAGGAGGTGTGTTAG
	*mPTBP1* forward	CAGCAACTCGGCAGCAAAC
	*mPTBP1* reverse	GCGTCACCGAGGTGTAGTAGTTC
Real-time PCR	*pPTBP1* forward	ACTTCCAGAACATCTTCCCACC
Primers	*pPTBP1* reverse	TTGAACCCTTTGACGATACCAC
	*ACTB* forward	TCCCTGGAGAAGAGCTACGA
	*ACTB* reverse	AGCACTGTGTTGGCGTACAG
	*pGAPDH* forward	ATGGATGACGATATTGCTGCGCTC
	*pGAPDH* reverse	TTCTCACGGTTGGCTTTGG
siRNA sequences	*si-PTBP1* sense	GCAACGGUGGUAUCGUCAATT
	*si-PTBP1* antisense	UUGACGAUACCACCGUUGCTT
	*si-MyD88* sense	GUACAAGGCAAUGAAGAAATT
	*si-MyD88* antisense	UUUCUUCAUUGCCUUGUACTT
	*si-TRAF3* sense	GGCCGUUUAAGCAGAAAGUTT
	*si-TRAF3* antisense	ACUUUCUGCUUAAACGGCCTT
	*si-TRAF6* sense	GCGCUGUGCAAACUAUAUATT
	*si-TRAF6* antisense	UAUAUAGUUUGCACAGCGCTT
	NC sense	UUCUCCGAACGUGUCACGUTT
	NC antisense	ACGUGACACGUUCGGAGAATT

### Cells and transfections

HEK 293T cells (ATCC, CRL-11268), African green monkey kidney (Vero) cells (ATCC, CCL-81), and porcine kidney 15 cells (ATCC, CCL-33) were cultivated in Dulbecco's modified Eagle's medium (D6429, Sigma-Aldrich) containing 10% fetal bovine serum (10099141, Gibco). LLC-PK1 cells were procured from Dr Rui Luo (Huazhong Agricultural University) and maintained in Minimum Essential Medium (11095080, Invitrogen). The above cell lines were incubated at 37 °C and 5% CO_2_. Cells were transfected at approximately 80% to 90% confluence with plasmids using Lipofectamine 3000 Reagent (L3000015, Invitrogen) according to specific protocols. Lipofectamine RNAiMAX (13778150, Invitrogen) was used to transfect cells with siRNA when 50% to 60% density was achieved.

### PEDV infection

The JS-2013 variant strain of PEDV was isolated and preserved in the laboratory for this study (31). For PEDV infection, Vero cells with more than 90% adherence in culture plates were rinsed with phosphate buffered solution (PBS) (C20012500BT, Gibco), followed by PEDV infection at (MOI = 1 or 0.01) and under trypsin (15050065, Invitrogen) treatment. The cells were rinsed with PBS after 1 h and cultured within serum-free Dulbecco's modified Eagle's medium containing trypsin for diverse periods at 37 °C prior to collection. The Spearman–Kaerber approach was utilized to calculate the viral titers, which were denoted as TCID_50_/ml.

### RNA isolation and qRT-PCR

RNeasy Mini Kit (74104, Qiagen) was employed to extract total RNA. Complementary DNA was then prepared using the PrimeScript RT reagent Kit (RRO47A, Takara Bio Inc) through reverse transcription. The SYBR Premix Ex Taq TM (q711–03, Vazyme Biotech Co., Ltd) was utilized for qRT-PCR. Table 1 lists primers utilized in qRT-PCR. All data were normalized to β-actin.

### Western blot assay

After rinsing with prechilled PBS, the cells were subjected to 5-min ice-cold incubation using the RIPA Lysis and Extraction Buffer (89901, Thermo Fisher Scientific) consisting of the Protease Inhibitor Cocktail (Bimake, B14001). The lysates were denatured using 5 X SDS-PAGE sample buffer for 10 min. The proteins were then separated through SDS-PAGE and transferred onto nitrocellulose membranes (10600001, GE Healthcare) for Western blot assay. The membranes were blocked using PBS containing 5% nonfat dry milk (232100, BD Biosciences) and 0.2% Tween 20 (Sigma-Aldrich, P1379). The membranes were further incubated with primary antibody at room temperature, followed by incubation with HRP-labeled secondary antibody. The proteins were detected by enhanced chemiluminescence (SB-WB012, Share-Bio).

### Co-IP assay

The cells were lysed after 24 h of transfection with specific plasmids using the NP40 cell lysis buffer (FNN0021, Life Technologies) containing the Protease Inhibitor Cocktail. The lysates were centrifuged and incubated with Dynabeads Protein G bound to anti-Flag-antibody (10004D, Life Technologies). The lysates were washed with PBS containing 0.02% Tween 20 and resuspended using 50 mM glycine elution buffer (pH 2.8). Western Blotting assays were performed using corresponding antibodies.

### GST affinity–isolation test

The full-length PEDV N, MARCH8, NDP52, and PTBP1 genes were cloned into the pCold GST plasmid (3372, Clontech Laboratories, Inc) or pCold TF plasmid (3365, Clontech Laboratories, Inc). The plasmids were expressed in BL21 competent cells (C504–03, Vazyme Biotech Co, Ltd). The GST Tag Protein Interaction Pull-Down Kit (21516, Thermo Fisher Scientific Pierce) was used for protein interaction analysis in line with specific protocols. After elution, Western blot assay was performed for protein analysis.

### Confocal immunofluorescence assay

In this assay, cells were fixed using 4% paraformaldehyde (P6148, Sigma-Aldrich) and permeabilized using 0.1% Triton X-100 (Sigma-Aldrich, T9284). Then, 5% bovine serum albumin (9998, Cell Signaling Technology) was utilized to block the cells, which were later incubated for 1 h with the primary antibody. After rinsing thrice with PBS, the cells were incubated for 1 h with the fluorescently labeled secondary antibody as previously described (7). The cells were incubated for 5 min with 4',6-diamidino-2-phenylindole (DAPI; C1002, Beyotime Biotechnology) to achieve nuclear staining. The laser scanning confocal immunofluorescence microscope (Carl Zeiss) was utilized for observing fluorescence images.

### Luciferase reporter assay

Plasmids that encoded the target genes were transfected into the HEK 293T cells using Lipofectamine 3000. The cells were collected after 24 h, and the luciferase activity was measured using the Dual-Glo Luciferase Assay System (DL101, Vazyme Biotech Co, Ltd). Renilla luciferase served as an endogenous reference.

### Statistical analysis

Differences between the two groups were estimated by Student’s *t* test (two-sided) and visualized by GraphPad Prism 5 software (GraphPad Software, Inc). *p* < 0.05 indicated statistical significance. Data were obtained from three separate assays.

## Data availability

All data are contained within the manuscript.

## Conflict of interest

The authors declare that they have no conflicts of interest with the contents of this article.
